# How the Metal Ion Affects the ^1^H NMR Chemical Shift Values of Schiff Base Metal Complexes: Rationalization by DFT Calculations

**DOI:** 10.1021/acs.jpca.3c05653

**Published:** 2023-10-31

**Authors:** Valeria Butera, Luisa D’Anna, Simona Rubino, Riccardo Bonsignore, Angelo Spinello, Alessio Terenzi, Giampaolo Barone

**Affiliations:** Dipartimento di Scienze e Tecnologie Biologiche, Chimiche e Farmaceutiche, University of Palermo, viale delle Scienze Edificio 17, Palermo 90128, Italy

## Abstract

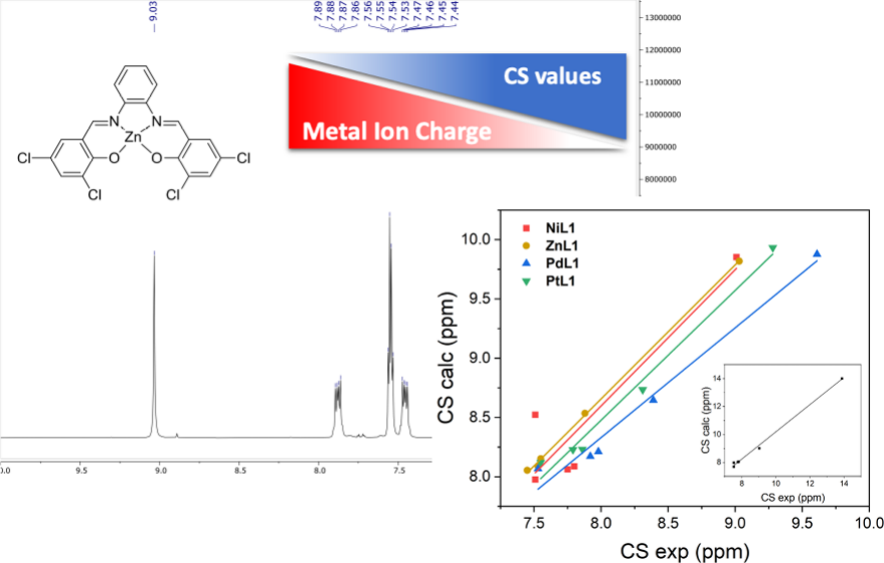

The chemical shift (CS) values obtained by ^1^H NMR spectroscopy for the hydrogen atoms of a tetradentate N_2_O_2_-substituted Salphen ligand (H_2_**L1**) are differently shifted in its complexes of nickel(II), palladium(II), platinum(II), and zinc(II), all bearing the same charge on the metal ions. To rationalize the observed trends, DFT calculations have been performed in the implicit *d*_6_-DMSO solvent in terms of the electronic effects induced by the metal ion and of the nature and strength of the metal-N and metal-O bonds. Overall, the results obtained point out that, in the complexes involving group 10 elements, the CS values show the greater shift when considering the two hydrogen atoms at a shorter distance from the coordinated metal center and follow the decreasing metal charge in the order Ni > Pd > Pt. This trend suggests a more covalent character of the ligand–metal bonds with the increase of the metal atomic number. Furthermore, a slightly poorer agreement between experimental and calculated data is observed in the presence of the nickel(II) ion. Such discrepancy is explained by the formation of stacked oligomers, aimed at minimizing the repulsive interactions with the polar DMSO solvent.

## Introduction

Nuclear magnetic resonance (NMR) spectroscopy is one of the leading analytical techniques to gain structural information on organic molecules and their possible metal complexes in solution.^1^ As NMR parameters are ground state properties, Density Functional Theory (DFT) is an excellent compromise, in particular in the presence of a metal, to quickly and reliably obtain ground state data for molecular systems of increasingly larger size.^2^ As with many other quantum chemical observables, both chemical shift (CS) and spin–spin coupling (SC) constants can be obtained at the increasingly higher levels of theory. This is particularly true for the CS parameter, while for the SC constants, the agreement with the experimental data proved to be more challenging.^3^ In fact, for a good agreement with the experimental data, spin–spin coupling constants need to be evaluated at the post-HF level.

DFT calculations have been successfully applied to investigate coordination compounds’ magnetic properties,^4^ particularly for ^1^H and ^13^C NMR CS values,^5−8^ obtaining, in general, a good agreement between calculated and experimental values, with a systematic error in the 10–20% range.

If we consider a molecular system, relativistic effects are important in the presence of a heavy metal ion, such as those of the second and third transition rows of the periodic table.^7,9^

Metal ion coordination to a ligand typically induces a shift in the NMR CS values of the light atoms, such as H and C, in diamagnetic compounds. A possible explanation of such phenomenon is provided by the heavy atom-light atom (HALA) effect.^10^ Indeed, the latter has been mainly ascribed to both scalar and spin–orbit relativistic effects of the heavy atom, and guidelines have been provided to interpret the NMR CS shift due to such HALA effect.^10^ However, looking for practical applications, such an effect is expected to play a big role only in the presence of 5d transition metals and strongly depends on the structure of the metal complex. For example, it depends on the HALA distance (e.g., if they are directly bonded or not) and on their relative (e.g., *trans* or *cis*) position. Moreover, it strongly depends on both HA and LA properties and on the nature of the HA-ligand bonds. For example, considering platinum as HA, both shielding and deshielding effects have been detected on the CS of NMR active nuclei, such as ^1^H, ^13^C, and ^15^N.^10^ These considerations show that the HALA effect is a phenomenon that is still not well understood and deserves to be deepened from a theoretical point of view.

**Figure 1:**
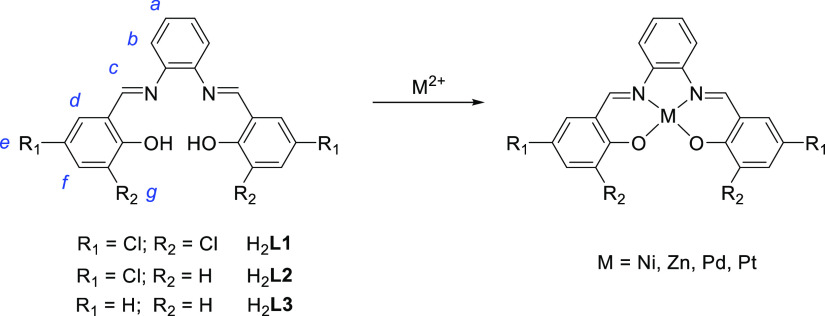
Structure and labeling of the considered metal complexes of ligand H_2_**L1** (M = Ni, Zn, Pd, Pt) and of the nickel complexes of ligands H_2_**L2** and H_2_**L3**.

**Figure 2:**
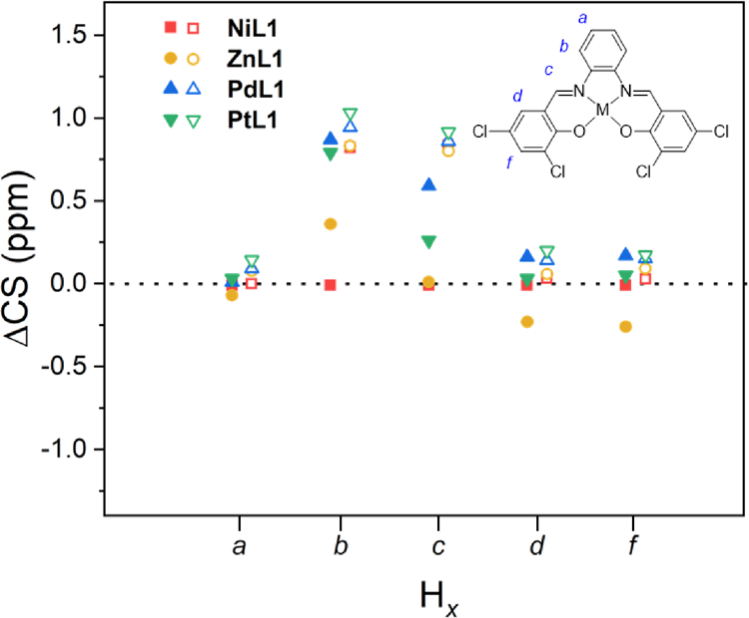
Graphical representation of the metal-induced shift of the experimental (filled symbols) and computed (empty symbols) CS values of protons H_X_ (X = a-d, f) of the four metal complexes compared to those of their ligand H_2_**L1**.

In the past few years, we have been involved in the synthesis and characterization of square planar Schiff base transition-metal complexes, also investigating their DNA-binding properties.^11−18^ In this context, we have recently reported on different metal complexes of a substituted Salphen ligand, H_2_**L1** in Figure 1.^19^ In these complexes, the metal ion is always charged 2+ and copper(II) is also included. However, due to its paramagnetic nature, its NMR spectra were not recorded. The compounds in Figure 1 showed interesting biological properties, including antiproliferative activity toward MCF-7, HepG2, and HeLa cell lines and a preferential binding affinity toward both c-KIT1 and c-KIT2 G-quadruplex DNAs. From a structural point of view, when recording ^1^H NMR spectra of the compounds, we noticed quite different CS values when Ni, Zn, Pd, or Pt ions were involved. This variation was particularly pronounced for atoms H_b_ and H_c_ (Figure 2), which are situated at a shorter distance from the metal ion. Intrigued by these results, in the following, we report the application of DFT methods to compare the experimental and calculated ^1^H NMR CS values of the above-mentioned complexes (Figure 1), providing a possible explanation to the observed trends and to the different behavior of the nickel(II) complex.

## Experimental Methods

### Synthesis and Characterization

Solvents and reagents (reagent grade) were all commercially available and used without further purification. All the compounds are literature known and were prepared according to or slightly modifying the reported procedures.^19^^1^H NMR and 2D ^1^H–^1^H COSY spectra were recorded in DMSO-*d*_6_ solution on a Bruker 400 MHz NMR spectrometer. UV–vis absorption spectra were collected on a Varian UV–vis Cary 1E double beam spectrophotometer, from DMSO solution of comparable concentration of nickel(II) complex **NiL1** and its ligand H_2_**L1**.

Ligand H_2_**L1** and its Pt, Pd, Ni, and Zn metal complexes, **PtL1**, **PdL1**, **NiL1**, and **ZnL1**, were synthesized as recently reported.^19^ Complexes **NiL2** and **NiL3** were prepared modifying or following the methods previously reported.^20^ In particular, **NiL2** was synthesized as follows:

**NiL2**: a solution of 5-chlorosalicylaldehyde (156.5 mg, 1 mmol) and 1,2-phenilendiamine (54.3 mg, 0.5 mmol) in ethanol was stirred for 30 min at room temperature before adding Ni(CH_3_COO)_2_·4H_2_O (136.6 mg, 0.55 mmol) previously dissolved in the minimum amount of H_2_O. The mixture was left stirring for 4 h. The resulting precipitate was washed with cold water, ethanol, and diethyl ether to afford **NiL2** as a red solid (106.4 mg, 48.1% yield).

To guide the reader through the ^1^H NMR analysis, representative spectra of the literature-known titled compounds are reported in the Supporting Information.

DMSO was selected as solvent for the spectroscopic characterization of H_2_**L1** and its Pt, Pd, Ni, and Zn complexes, since these compounds showed the highest solubility in this solvent, compared both to water and to less polar solvents, such as chloroform or acetonitrile.

### Computational Details

DFT calculations were performed using the GAUSSIAN-16^21^ and ORCA^22^ program packages. Different DFT functionals have been considered, including both pseudopotential and all-electron basis set and, as reported in the Supporting Information (Tables S1 and S2), preliminary tests were performed on the zinc(II) complex **ZnL1**, to select the best combination of DFT functional and basis set with the highest linear correlation coefficient of experimental vs calculated ^1^H NMR CS values. The results obtained point out that the difference between experimental and calculated CS values is mainly due to the basis set type rather than to the DFT functional (Table S1). For example, by using Pople basis sets such as 6-311G(d,p), an underestimation of the computed CS values occurs. On the other hand, by using the PCSSEG-2 basis set, an overestimation always occurs regardless of the functional used. Concerning the different DFT functionals considered, ω-B97X-D3 provides the smallest computed-experimental difference, which further decreases when the def2-TZVP basis set is employed. Geometry optimization of the investigated metal complexes was performed using the ω-B97X-D3^23^ functional, which includes Grimme’s dispersion correction,^24^ and Aldrich’s def2-TZVP^25^ basis set for all the atoms but Pd and Pt atoms, for which the def2-ECP is automatically set by ORCA. To speed up the calculations, the RIJCOSX^26^ resolution of identity approximation has been used, which includes the RI-J for the Coulomb integrals and COSX numerical chain-of-sphere integration for the HF exchange integrals. Moreover, the decontracted auxiliary def2/J Coulomb fitting basis sets have been employed as indicated in the ORCA manual.

Charges on the metal centers have been calculated by the CHelpG method.^27^^1^H NMR magnetic isotropic shielding constants (σ) were calculated by using the gauge-independent atomic orbital (GIAO) method.^28^ We performed single point calculations using the same basis sets as for the optimization (def2-TVZP along with the def2/J). The results were then compared with those obtained using the Zero Order Relativistic Approximation (ZORA)^29^ scalar relativistic method. In this case, the relativistically recontracted all-electron ZORA-def2-TZVP^30^ basis set was used for all the atoms, along with the SARC/J^31^ auxiliary basis set. Moreover, for the critical case of the Ni complex, ^1^H NMR chemical shifts of the Ni complex were recalculated using all-electron ANO-RCC-DZP, also employing the HF level of theory, together with the def2-TZVP def2/J basis set. The polarizable continuum model (CPCM)^32^ approach was used to describe the effects of the DMSO solvent, in which the experimental spectra were recorded.

The chemical shift (δ) values were determined on a scale relative to tetramethylsilane (TMS), as a reference, and calculated according to the following equation:
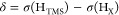
where H_X_ is one of the hydrogen atoms of the Schiff base ligand and where the isotropic shielding of the six equivalent hydrogen atoms of TMS, H_TMS_, was computed using the same methodology. Being the studied compounds of roughly C_2v_ point group symmetry, the averaged CS values of symmetrically equivalent protons are discussed throughout the text. The calculated CS values of each H atom of all of the considered compounds are reported in the Supporting Information (Tables S4–S11).

## Results and Discussion

The experimental CS values are reported in Table 1 and Figures S1–S5, while their shift compared to H_2_**L1** is graphically represented in Figure 2. As mentioned above, the analysis of this data set clearly demonstrates a distinctive impact of each coordinated metal atom on the ligand proton CS values. Specifically, the largest variation in chemical shifts occurs when heavier Pt and Pd metals are involved, followed by Zn, while Ni has a minimal effect on the ^1^H NMR CS of H_2_**L1**. Pd and Pt, for instance, induce downfield shifts of +0.9 and +0.8 as well as +0.6 and +0.3 ppm, respectively, to the two closest protons to the metal center, H_b_ and H_c_, respectively (Figure 2). Conversely, H_a_, being furthest from the metal center, appears to remain essentially unaffected by the presence of any metal, resulting in negligible shifts compared to the free ligand H_2_**L1**. Thus, excluding H_a_, a general trend could be however observed for the remaining protons (Figure 2); while the heavier atoms induce downfield shifts to all the remaining protons, Zn^2+^ (yellow circles in Figure 2) causes such a shift only for H_b_, with H_c_ CS remaining almost unaltered and H_d_ and H_f_ CS values showing a slight upfield shift. Moreover, when a Ni^2+^ ion is bound to H_2_**L1** (represented as red squares in Figure 2), minimal variations are observed in the CS values of all protons.

**Table 1:** Experimental and Calculated (at the ω-B97X-D3/def2-TZVP Level, def2-ECP Basis Set for Pd and Pt) ^1^H NMR CS (ppm), Obtained in DMSO for the Ligand H_2_**L1** and the Four-Considered Metal Complexes^a^

	**H**_**2**_**L1**		**NiL1**		**ZnL1**		**PdL1**		**PtL1**	
H label	exp	calc	exp	calc	exp	calc	exp	calc	exp	calc
a	7.52	7.98	7.51	7.98	7.45	8.06	7.53	8.07	7.55	8.12
b	7.52	7.70	7.51	8.52	7.88	8.54	8.39	8.65	8.31	8.73
c	9.02	9.02	9.01	9.85	9.03	9.82	9.61	9.88	9.28	9.93
d	7.76	8.03	7.75	8.06	7.53	8.09	7.92	8.17	7.79	8.23
f	7.81	8.06	7.8	8.09	7.55	8.15	7.98	8.21	7.86	8.23
OH	13.88	14.02								
***q***				0.50		1.08		0.34		0.24
*R*		0.9983		0.9172		0.9995		0.9884		0.9919

a The calculated metal charge ***q*** (au) and linear correlation coefficient *R* of each linear fitting are also reported.

To rationalize the experimental results, we carried out DFT calculations on the four metal complexes and on H_2_**L1**. The structure of H_2_**L1** (Figure S10) is not planar, with the torsion angle involving the two dichlorophenol moieties rotated by ∼50° with respect to the plane of the third diimine bearing ring. On the other hand, the optimized structures of all four-substituted Salphen metal complexes confirm the expected N_2_O_2_ tetra coordination in a roughly square planar geometry (Figure S11). Detailed structural analysis and comparison with available experimental data are reported in the Supporting Information.

The experimental and theoretical CS values of the protons of each complex are reported in Table 1. In all considered cases, the calculated CS values are systematically larger than the corresponding experimental data. According to both the experimental and computational results, the most deshielded proton for all of the studied complexes is H_c_, resonating at frequencies higher than 9 ppm (see also Figure 2). This effect is enhanced in the presence of Pd and Pt, for which the largest CS values are obtained. On the other hand, the H_a_ protons are less affected by the presence of the metal, presumably due to their largest distance from the metal center, leading to smaller CS values.

**Figure 3:**
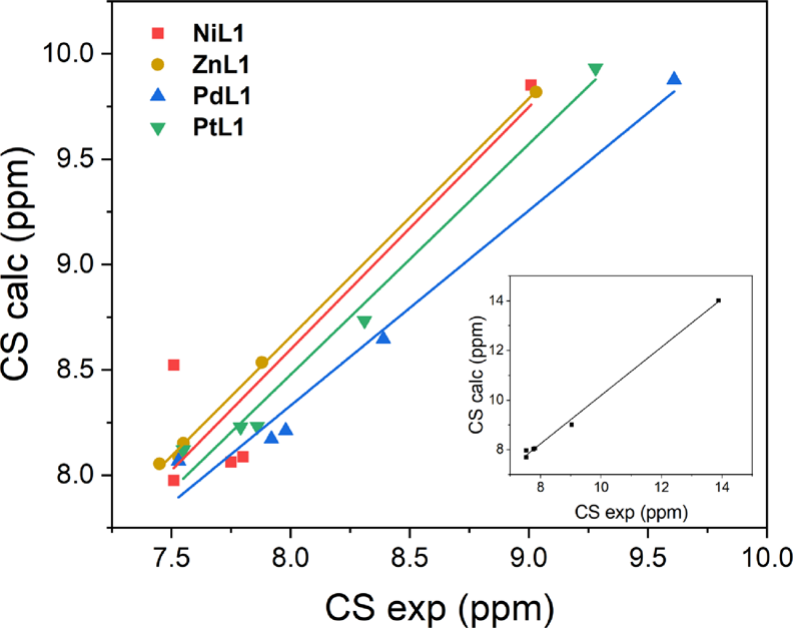
Linear correlation plot between calculated and experimental CS values, for the ligand (H_2_**L1**, inset) and the four-considered metal complexes (M = Ni, Zn, Pd, Pt). Corresponding linear fittings are indicated with the same color.

The experimental CS values of H_a_ for all of the considered complexes are in the range 7.45–7.55 ppm, while the corresponding calculated values are increased to 7.98–8.12 ppm. For all complexes, the experimental and theoretical CSs of H_d_ and H_f_ protons are very similar, with differences lower than 0.07 ppm, underlying the idea that, despite their different locations, those protons experience a similar shielding effect from their chemical environment. DFT calculations support the highest shielding effect of Pt and Pd heavy metals on H_b_ protons, followed by H_d_ and H_f_ with respect to the H_2_**L1** ligand. Interestingly, for all of the complexes but **NiL1**, both the calculated and experimental CS values of H_b_ lie at a lower field in comparison to those of the corresponding H_d_ and H_f_ protons. For the Ni complex, the experimental results underline an inversion of the trend, with the CS of the H_b_ proton falling at a lower frequency of 0.3 ppm than those of H_d_ and H_f_. This behavior is not supported by DFT calculations, based on which the trend of all the chemical shifts of the Ni complex is identical to the other studied complexes. Thus, a generally strong linear correlation was observed between the calculated and experimental CS values for the ligand H_2_**L1** and three of the considered metal complexes (see Figure 3).

**Figure 4:**
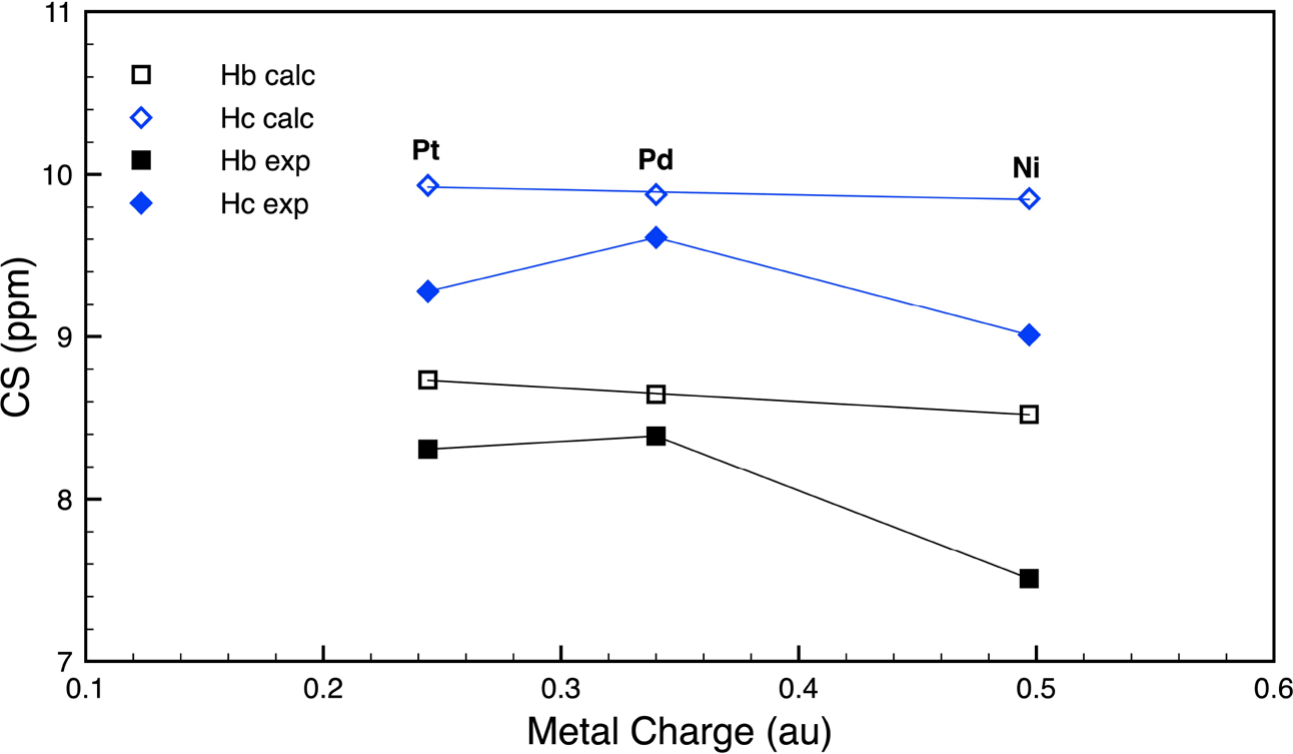
Linear correlation plot between calculated (empty symbols) and experimental (filled symbols) CS values of H_b_ and H_c_ vs the charge of the metal, for **NiL1**, **PdL1**, and **PtL1**.

Interestingly, if we restrict our analysis to the metal complexes of group 10 elements, all of which share the same d^8^ external electron configuration, the increase of the ^1^H NMR CS value calculated for both H_b_ and H_c_ follows the order Pt > Pd > Ni. This trend nicely mirrors a decrease in the charge of the metal ion following the opposite sequence, Ni > Pd > Pt (Figure 4, empty symbols). Such observation can be related to the increase of the covalent nature, in particular of the M-N bonds, progressing in the order Pt > Pd > Ni. A Natural Bond Orbital (NBO) analysis, carried out on the **PtL1** complex, Figure S13, shows that the metal-O and metal-N bonds can be essentially described as sigma bonds. The covalent nature of the bond significantly affects the deshielding of the nearest neighboring H nuclei, connected through electron conjugated bonds. The inversion of the experimental trend observed with Pt and Pd for both H_b_ and H_c_ is indeed interesting to note (Figure 4, filled symbols). In fact, an analogous trend of downfield shifts is known to occur in the ^1^H NMR CS for the metal-bonded H atom of transition-metal hydride complexes of group 10, with the same Pd > Pt > Ni order.^33^ The theoretical reproduction of such an effect requires a fully relativistic treatment, including spin–orbit coupling. The trend second row > third row > first row elements in the ^1^H NMR CS downfield shift has been observed for groups 6–10, all characterized by partially filled valence d orbitals.

The data plotted in Figure 3 and reported in Table 1 show that a major discrepancy occurs for the **NiL1** complex, resulting in a consistently lower experimental-theoretical linear correlation. The experimental CS values were validated via two-dimensional ^1^H–^1^H COSY NMR (Figure S6), thus excluding any possible error in the compound peak assignments. Furthermore, the UV–vis absorption spectra of both **NiL1** and H_2_**L1**, collected in DMSO (Figure S7), confirmed the retention of the metal center in the presence of the solvent, thereby excluding its hydrolysis during the NMR experiments.

**Figure 5:**
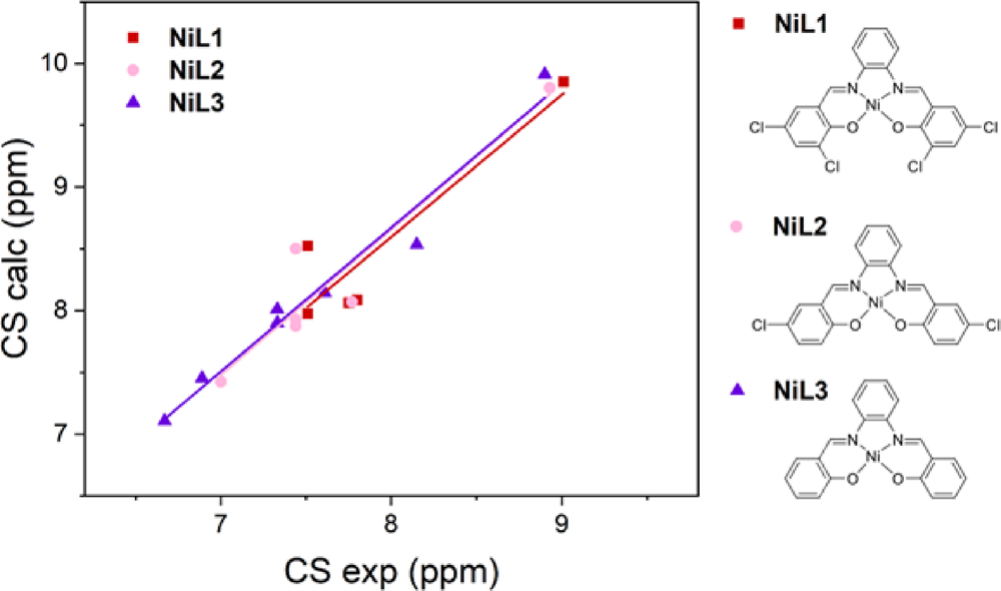
Linear correlation plot between calculated and experimental CS values for the three nickel(II) complexes of ligands H_2_**L1**, H_2_**L2**, and H_2_**L3**. Corresponding linear fittings are indicated with the same color.

The observed effect was then attributed to the presence of the Cl substituents in the aromatic rings. To corroborate such hypothesis, the analogous nickel(II) complexes of Salphen ligands H_2_**L2** and H_2_**L3** were also prepared (Figure 1; experimental ^1^H NMR spectra in Figures S8 and S9), thus allowing a comparison of the CS values in the presence of four (H_2_**L1**), two (H_2_**L2**), and no chlorides (H_2_**L3**) in the nickel complexes. Computed vs experimental data are shown in Table 2 and Figure 5 and highlight that the elimination of the chloride substituents gradually improves the experimental vs theoretical linear correlation of the corresponding CS values. In fact, the correlation coefficient values are 0.9172, 0.9431, and 0.9838, for **NiL1**, **NiL2**, and **NiL3**, respectively.

**Table 2:** Experimental and calculated ^1^H NMR CS, obtained in DMSO for the nickel(II) complexes of H_2_**L2**, H_2_**L3**, and of the stacked dimer **(NiL1)**_**2**_, at the ω-B97X-D3/def2-TZVP level^a^

	**NiL2**		**NiL3**		**(NiL1)**_**2**_**H**_**a**_**-H**_**f**_		**(NiL1)**_**2**_**H**_**a**_′-H_**f**_′	
H label	exp	calc	exp	calc	exp	calc	exp	calc
a	7.44	7.93	7.33	7.90	7.51	7.97	7.51	8.00
b	7.44	8.50	8.15	8.54	7.51	8.17	7.51	8.61
c	8.93	9.80	8.90	9.91	9.01	9.10	9.01	9.55
d	7.77	8.07	7.61	8.15	7.75	7.94	7.75	6.65
e			6.67	7.11				
f	7.44	7.88	7.33	8.01	7.80	8.21	7.80	7.80
g	7.00	7.42	6.89	7.45				
*R*		0.9431		0.9838		0.9536		0.6347

a The linear correlation coefficient *R* of each linear fitting is also reported.

**Figure 6:**
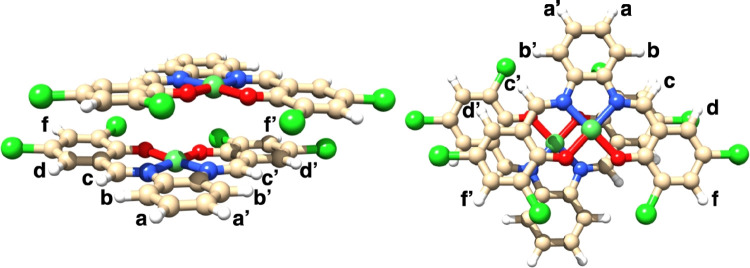
Stacked dimer of the nickel(II) complex of H_2_**L1**, **(NiL1)**_**2**_, and proton labels, whose structure has been obtained at the ω-B97X-D3/def2-TZVP level. Side (left) and top (right) views.

We have attributed such a finding to the increase in the lipophilic nature of the nickel complex as the number of chlorine atoms increases. The lipophilic nature of **NiL1**, in the polar DMSO solvent, could give rise to the formation of stacked aggregates. Ni-Salphen stacked complexes in the solid state have been recently reported,^20^ and interestingly, all the investigated complexes assume an antiparallel stacking arrangement in the crystal array. Starting from the crystal structure of Ni-MeOSalphen stacked dimers, we optimized the structure of **(NiL1)**_**2**_ (Figure 6).

**Figure 7:**
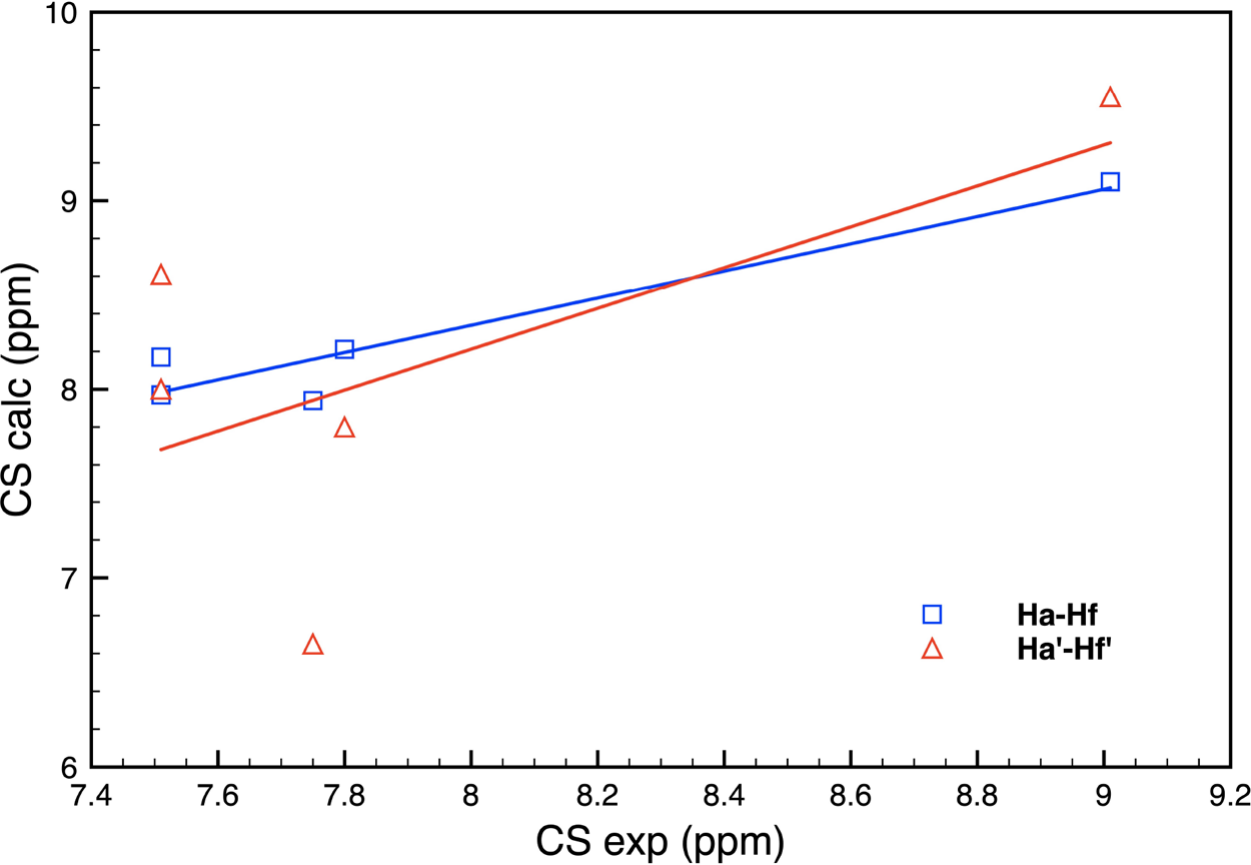
Linear correlation plot between calculated and experimental CS values for nuclei H_a_-H_f_ and H_a_′-H_f_′ of **(NiL1)**_**2**_. Corresponding linear fittings are indicated with the same color.

Such dimer is characterized by a roughly C_i_ point group symmetry and two sets of equivalent H atoms (labeled a-f and a′-f′, respectively) are found, whose corresponding CS values have been averaged (see Table S11). The calculated vs experimental linear correlation plot is reported in Figure 7. Notably, the calculated CS values for the a-f labeled hydrogen atoms (Table 2, last columns) in **(NiL1)**_**2**_ show a sensibly improved correlation of 0.9536 with the corresponding experimental data compared to those in **NiL1** (0.9172). This observation supports the tendency of apolar Ni complexes to preferentially form stacked aggregates in polar solvents, such as DMSO. By looking at both the crystal structure of the Ni-MeOSalphen dimer and the optimized structure of **(NiL1)**_**2**_, it can be noted that, presumably to improve the π–π stacking interactions, the bottom and top complexes adopt a slight offset orientation with respect to internuclear Ni–Ni axis.

Therefore, experimentally, an average value of the four analogous H nuclei should be obtained. The calculated values of protons H_b_ and H_a_ exhibit a greater similarity, in better agreement with the experimental values, which are indeed identical. Similarly, the CS value of the H_c_ proton in the stacked **(NiL1)**_**2**_ complex agrees well with the experimental one. However, the experimental-theoretical linear correlation observed for a′-f′ labeled H atoms of **(NiL1)**_**2**_ is poorer (R = 0.6347). The main reason for such disagreement is attributable to the calculated CS value of H_d_′, to the structural sensitivity of the CS values, and how they are affected by the local environment. H_d_′ is in fact at a larger distance from the two Cl atoms of the stacked complex, both at about 4.5 Å. In contrast, the H_d_ nucleus is 3.3 Å from the nearest Cl atom of the stacked **NiL1** complex. This result is the consequence of a side shift of the two-stacked **NiL1** complexes occurring during the geometry optimization process. Additionally, in the starting crystallographic structure, the two complexes are axially stacked with equal interatomic distances of symmetrically equivalent atoms. Such a result is, in our opinion, attributable to the limited dimer model of the stacked polymer in solution. Possibly, a longer stacked oligomer is present in solution, whose DFT modeling is however challenging and was not considered in the present work. However, the discrepancy between computed and experimental data could also be affected by the geometrical variability of the dimer, due to the flexibility of the Salphen ligand.

Finally, it is worth noting that the best correlation obtained for the stacked **(NiL1)**_**2**_ structure is still lower than those obtained for the mono Zn, Pt, and Pd complexes. On the other hand, less accurate agreement in nickel complexes has been already reported by Kondrashova et al.^8^ In their work, the authors report a comparison between calculated and experimental ^13^C NMR CS values of 157 nickel compounds, analyzing the performance of several DFT functionals and basis sets. A linear correlation plot of the calculated vs the experimental CS values allows one to obtain a root-mean-square error (RMSE) in the range 4.0–4.6 ppm, which increases up to 6.4 ppm for the Ni complexes. Interestingly, it is reported that the use of extended basis sets (such as the TZV used in this work) is critical also in the geometry optimization procedure, for a more reliable description of the electronic structure, in particular in complexes with multiple C–C bonds.

## Conclusions

DFT calculations were carried out in an attempt to rationalize the chemical shift (CS) values acquired through ^1^H NMR spectroscopy for the hydrogen atoms of a tetradentate N_2_O_2_ Cl-substituted Salphen ligand, H_2_**L1**, which displays distinct shifts in its complexes with nickel(II), palladium(II), platinum(II), and zinc(II), despite all metal ions carrying the same charge. In summary, as expected, the nearest protons to the metal center, H_b_ and H_c_, are the most deshielded in all metal complexes. The effect is enhanced in the presence of the heavier Pd and Pt metals, for which the largest experimental and theoretical CS values were obtained. On the other hand, the H_a_ protons are the less affected by the presence of the metals due to their furthest position from the metal center. For each complex, the experimental and theoretical chemical shifts of H_d_ and H_f_ protons are very similar, with differences within 0.07 ppm, underlying an analogous shielding effect of the different metals. The calculated larger shift of the H_b_ and H_c_ nuclei nicely correlates with the reduction in the metal ion charge upon going from Ni to Pd and finally to Pt. This result suggests that, within complexes featuring group 10 elements, both the metal ion electron density and metal–ligand bond covalency affect the electron density of the nearest neighboring H nuclei. The larger downfield shift experimentally observed for both H_b_ and H_c_ nuclei with Pd compared to Pt deserves a further investigation by using fully relativistic DFT calculations, as revealed by a theoretical study on transition-metal hydride complexes of group 10.^33^ The comparison between experimental and calculated ^1^H NMR CS values shows a generally high linear correlation. The lower correlation obtained for the nickel(II) complex **NiL1** opens a discussion on the actual structure adopted by this compound in DMSO. The presence of four chlorine atoms in the substituted Salphen ligand leads to the suggestion that a stacked polymer is present in solution, in an antiparallel arrangement as suggested by solid state structural studies,^20^ to reduce the contacts between the apolar complex and highly polar solvent.

## Supporting Information Available

Experimental ^1^H NMR spectra of the investigated ligand and metal complexes, computational methods tests performed on the zinc(II) complex, structural details of the model systems obtained by DFT calculations, values of all the calculated ^1^H NMR values and Cartesian coordinates for all considered systems (PDF)
