# Supplementary material for: How the Metal Ion Affects the 1H NMR Chemical Shift Values of Schiff Base Metal Complexes: Rationalization by DFT Calculations
Source: J Phys Chem A. 2023 Oct 31;127(44):9283–90. doi: 10.1021/acs.jpca.3c05653 (PMC10641838; doi:10.1021/acs.jpca.3c05653)
Supplement: Supplementary file 1 — jp3c05653_si_001.pdf [file jp3c05653_si_001.pdf]

How the metal ion affects the ^{1}H-NMR chemical shift values of Schiff base metal complexes: rationalization by DFT calculations

*Valeria Butera[#], Luisa D'Anna[#], Simona Rubino, Riccardo Bonsignore, Angelo Spinello, Alessio Terenzi and Giampaolo Barone**

Dipartimento di Scienze e Tecnologie Biologiche, Chimiche e Farmaceutiche, University of Palermo, viale delle Scienze Edificio 17, 90128 Palermo, Italy.

[#] These authors contributed equally

Supporting Information

Table of content:

1. **Spectroscopic characterization of H_2L1, NiL1, ZnL1, PdL1, PtL1, NiL2 and NiL3**

2. **Preliminary tests on ZnL1 to select the computational method**

3. **Optimized structures of H_2L1, NiL1, PdL1 and PtL1**

4. **Calculated ^{1}H-NMR CS values of the investigated compounds**

5. **Cartesian coordinates (Å) of the investigated compounds**

1. Spectroscopic characterization of H_2L1, NiL1, ZnL1, PdL1, PtL1, NiL2 and NiL3

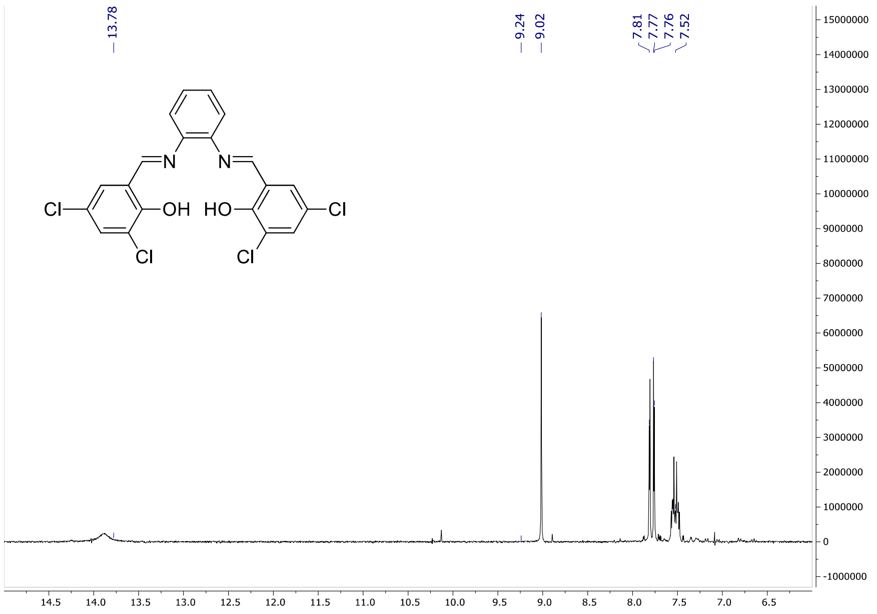

Figure S1. ^{1}H-NMR spectrum of H_2**L1**

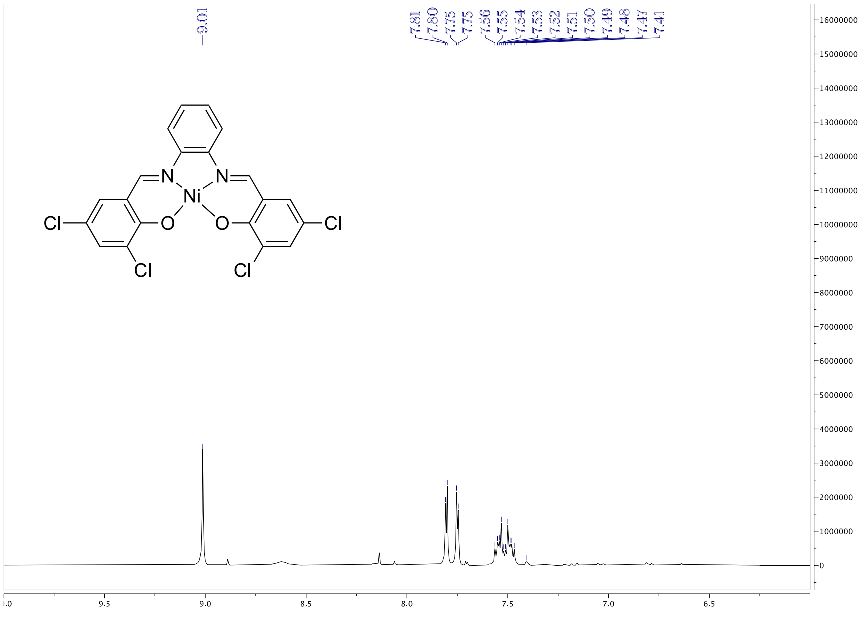

Figure S2. ^{1}H-NMR spectrum of **NiL1**

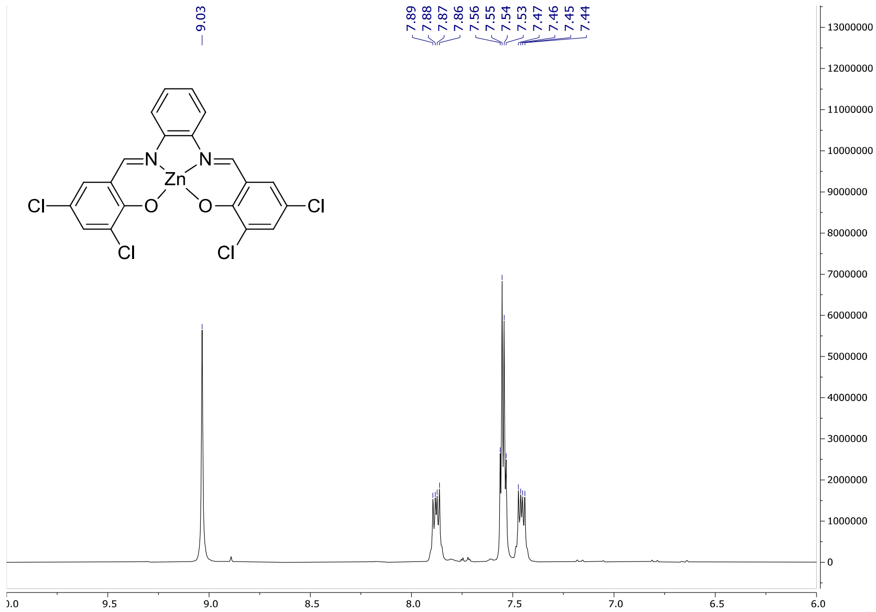

Figure S3. ^{1}H-NMR spectrum of **ZnL1**

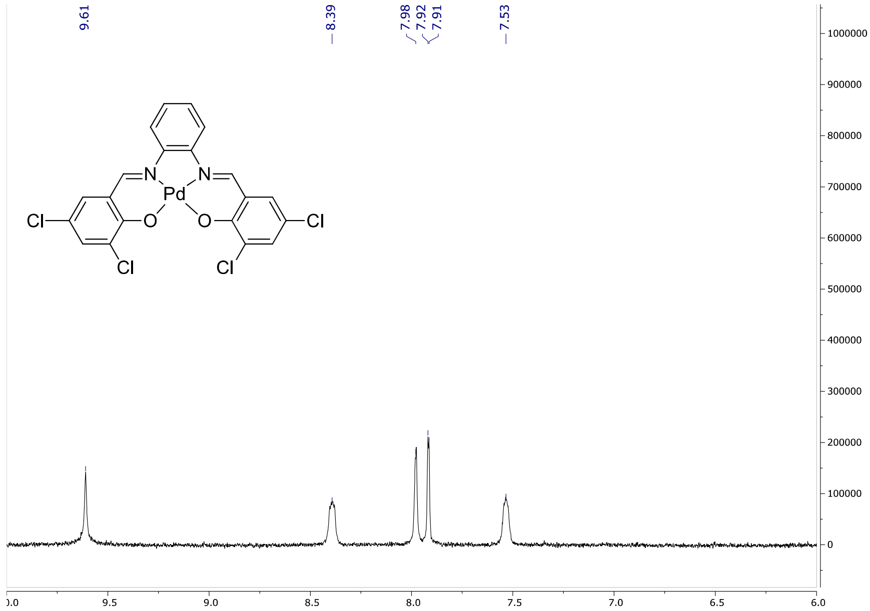

Figure S4. ^{1}H-NMR spectrum of **PdL1**

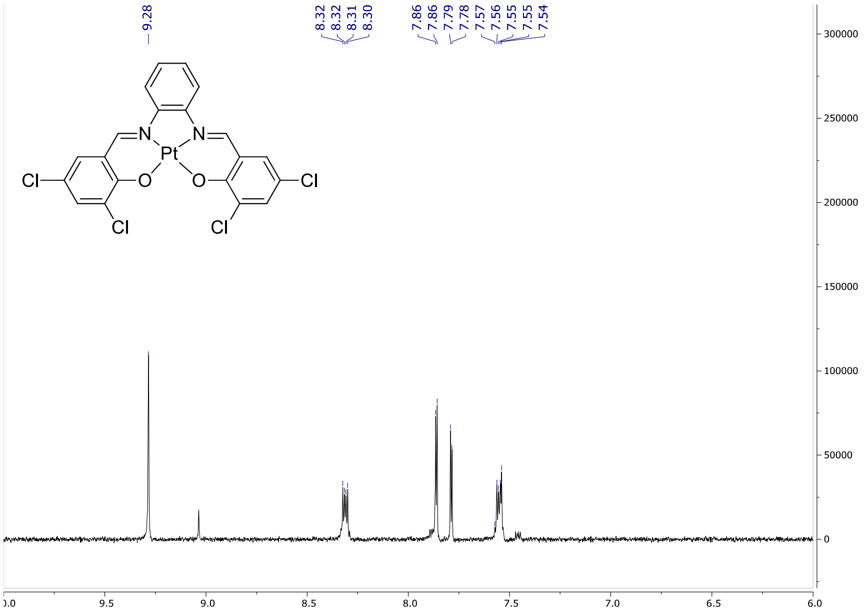

Figure S5. ^{1}H-NMR spectrum of **PtL1**

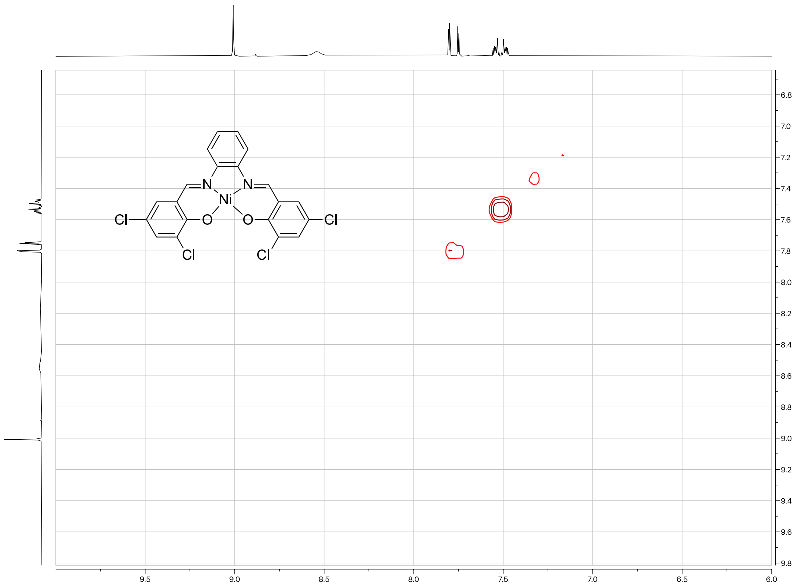

Figure S6. ^{1}H-NMR COSY spectrum of **NiL1**

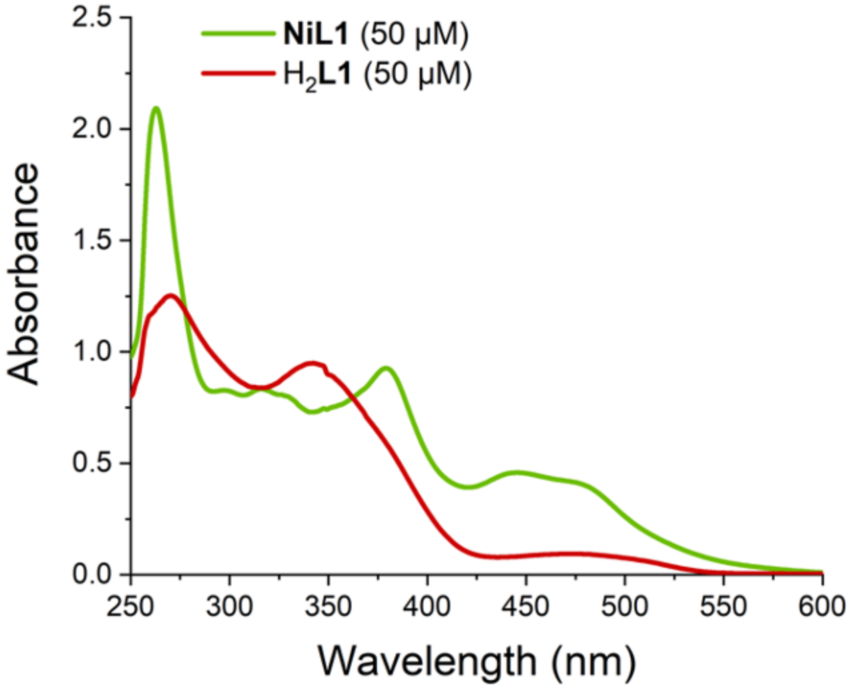

Figure S7. UV-Vis spectra of **NiL1** and H_2**L1**

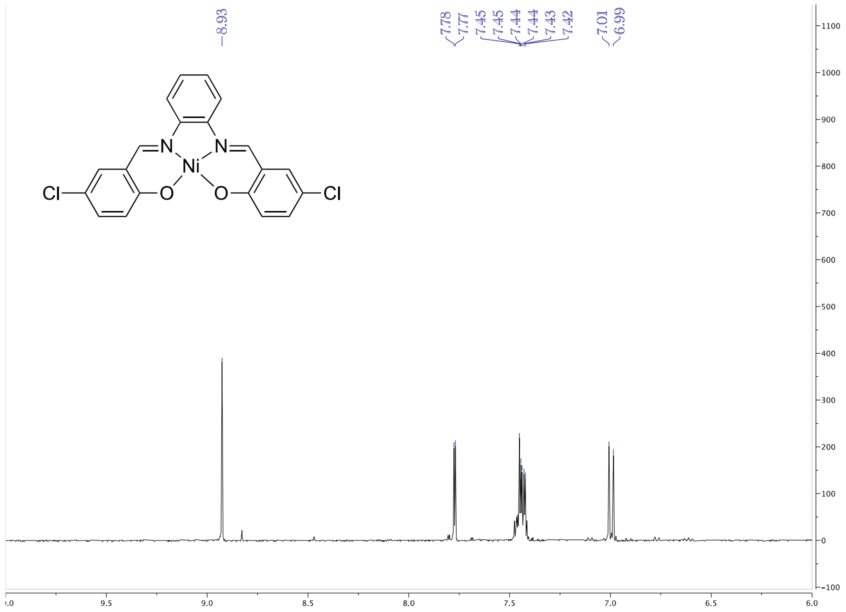

Figure S8. ^{1}H-NMR spectrum of **NiL2**

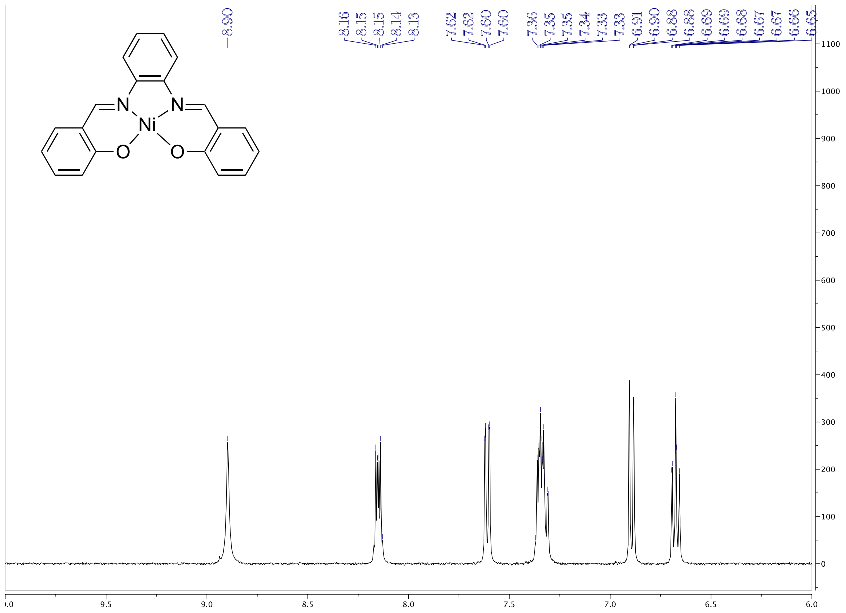

Figure S9. ^{1}H-NMR spectrum of **NiL3**

2. Preliminary tests on ZnL1 to select the computational method

In order to select the best combination of DFT functional and basis set which lead to the highest linear correlation coefficient, we performed several tests on the **ZnL1** complex. We first optimized the complex structure at the M06L level of theory with 6311G(d,p) basis set for all atoms but Zn, for which the LANL2DZ ECP was employed. We then calculate the values of the proton chemical shifts using the same protocol, obtaining an experimental vs calculated linear correlation coefficient of 0.9674. We checked if different combination of functionals and basis sets could result in an improvement of the correlation. Therefore, starting from the structure optimized with M06L functional, the ^{1}H-NMR CS values were calculated employing the all-electron 6-311G** basis sets with M06L, PBE0, B3LYP and ω-B97XD, whose linear correlation coefficients are reported in Table S1. Interestingly, the extension of all-electron basis sets to the metal cation leads to a worsening of the R value for both M06L and PBE0, while the obtained values at the B3LYP level of theory are almost identical. On the other hand, a better linear correlation is obtained when ω-B97XD is used, underlying the notable improving contribution of the dispersion forces included in this functional. We tested the performance of the same functionals in combination with the Ahlrichs def2-TZVP basis

set for the optimization of the structures. In this case, the ORCA software was used, to include the possibility of using RI integrals. ^{1}H-NMR CS values were also calculated using the PCSSEG-2 basis set, which is suited for NMR calculations, as reported in ORCA manual. The corresponding linear correlation coefficients are listed in Table S2, and clearly show the better performance of the ω-B97XD functional. We eventually checked the employment of def2-TZVP to further improve the experimental/calculated linear correlation.

Table S1. Calculated ^{1}H-NMR CS values (ppm) and linear correlation coefficients of **ZnL1** using different computational protocols with Gaussian06. The isotropic shielding of the TMS reference calculated at each level of theory is also reported (au)

	Exp	M06L/LANL2DZ/ 6-311G(d,p)	M06L/ 6-311G(d,p)	PBE0/ 6-311G(d,p)	ω-B97XD/ 6-311G(d,p)	B3LYP/ 6-311G(d,p)
**a**	7.45	7.58	7.57	7.77	7.83	7.66
**b**	7.88	7.86	7.82	8.02	8.03	7.91
**c**	9.03	8.99	8.92	9.01	9.18	8.94
**d**	7.53	7.18	7.14	7.40	7.51	7.29
**f**	7.55	7.35	7.31	7.54	7.62	7.45
**TMS**		32.20	32.20	31.83	31.98	31.99
**R**		0.9674	0.9633	0.9637	0.9750	0.9666

	Exp	M06L/PCSSEG-2	PBE0/PCSSEG-2	ω-B97XD/6/PCSSEG-2
**a**	7.45	7.72	7.98	8.07
**b**	7.88	8.15	8.48	8.56
**c**	9.03	9.30	9.63	9.78
**d**	7.53	7.57	7.87	8.07
**f**	7.55	7.79	8.07	8.18
**TMS**		32.15	31.46	31.60
**R**		0.9912	0.9924	0.9983

Table S2. Calculated ^{1}H-NMR CS values (ppm) and linear correlation coefficients of **ZnL1** using different computational protocols with ORCA. The isotropic shielding of the TMS reference calculated at each level of theory is also reported (au).

	Exp	M06L/ PCSSEG-2	PBE0/ PCSSEG-2	ω-B97XD/ PCSSEG-2	ω-B97XD/ def2-TZVP
**a**	7.51	7.72	7.98	8.07	8.05
**b**	7.51	8.15	8.48	8.56	8.53
**c**	9.01	9.30	9.63	9.78	9.82
**d**	7.75	7.57	7.87	8.07	8.09
**f**	7.8	7.79	8.07	8.18	8.15
**TMS**		32.15	31.46	31.61	31.87
**R**		0.9912	0.9924	0.9983	0.9990

3. Optimized structures of H_2L1, NiL1, PdL1 and PtL1

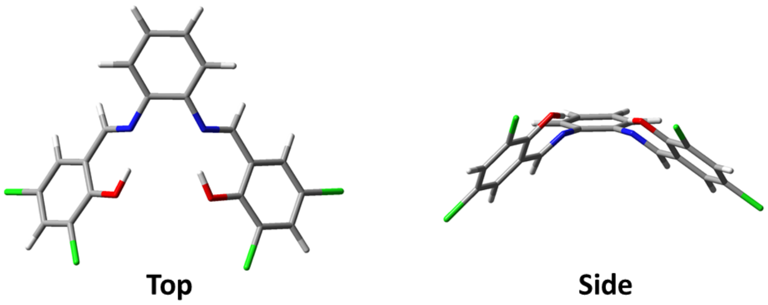

Figure S10. Top and side view of H_2**L1**

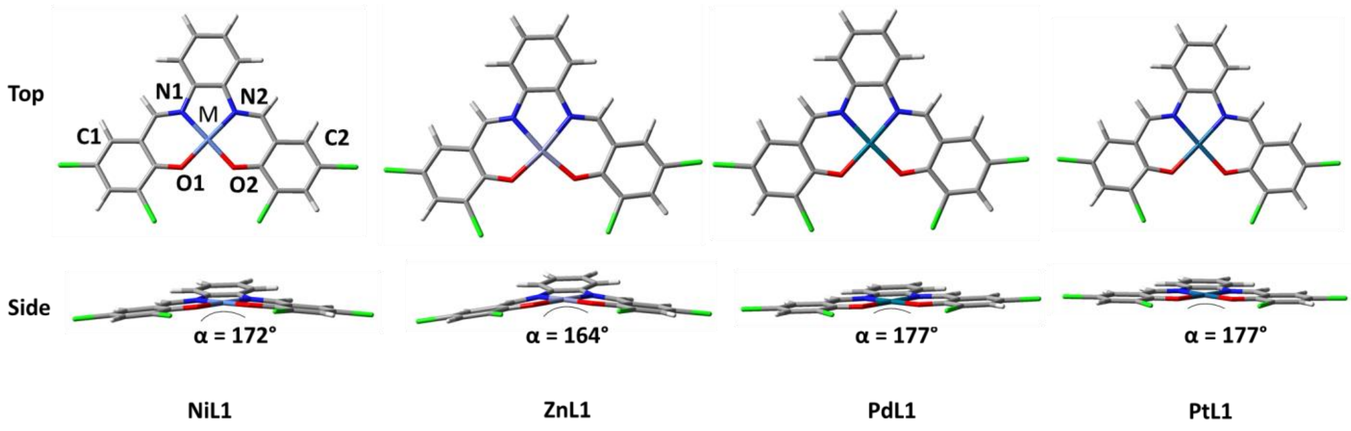

Figure S11. Top and side view of the optimized structures of **ML1** (M = Ni, Zn, Pd and Pt). The calculated values of the C1─M─C2 angle are also shown.

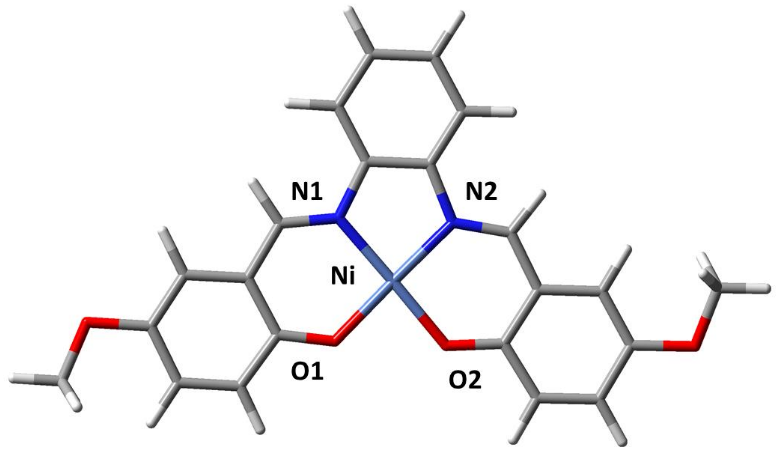

Figure S12. Structure of Ni-MeOSalphen complex reported in ref 20.

Table S3. Main calculated geometric parameters of **NiL1** (distances, Å and angles, °) and corresponding experimental values of the Ni-MeOSalphen complex reported in ref. 20.

	Experimental	Calculated
Ni-N1	1.8626(12)	1.86725
Ni-N2	1.8643(13)	1.86815
Ni-O1	1.8536(11)	1.85313
Ni-O2	1.8394(11)	1.85311
O1-Ni-N2	177.72(5)	178.91034
O1-Ni-N2	95.22(5)	94.50216

The structure of H_2**L1** (Figure S10) is not planar, with the torsion angle involving the two dichlorophenol moieties rotated of ~50° with respect to the plan of the third diimine bearing ring. On the other hand, the optimized structure of all the four substituted Salphen metal complexes confirm the expected N_2O_2 tetra coordination in a roughly square planar geometry (Figure S11). The most stable optimized structures of H_2**L1**, (Figure S10), reveals the formation of two hydrogen bonds between the hydrogen atoms of the hydroxyl groups and the N atoms, whose calculated distance is 1.71 Å. Table S3 reports the main geometric parameters calculated for complex **NiL1** (Figure S11) and the experimental ones reported in ref. 20 for a similar Ni-MeOSalphen complex (Figure S12). The very good agreement between the calculated and experimental values nicely supports the reliability of the selected computational protocol. The calculated O1-N1-N2-O2 dihedral angle is -0.86° while the C1-Ni-C2 is 172.0°. When Ni is replaced by Zn, the calculated O1-N1-N2-O2 and C1-Ni-C2 angles become 0.00° and 164.9°, respectively, which results in a reduced planarity of the aryl halides substituents while the Zn cation reaches a perfect squared planar coordination. Similarly to **ZnL1**, for both **PdL1** and **PtL1** the calculated O1-N1-N2-O2 angle is 0.00°, while the C1-Ni-C2 angle values are 176.6° and 176.8°, respectively. Therefore, the presence of different metal ions affects the bending of the two aryl halide groups inferring a distortion from the planarity to the overall structure. Such an effect is more pronounced in the case of **ZnL1**, while it is almost negligible in the presence of the heavier Pt and Pd metals. Interestingly, only for the **NiL1** the calculated O1-N1-N2-

O2 dihedral angle is different from zero. The M-N distances decrease from 2.06 Å in **ZnL1** to 1.87, 1.97 and 1.96 Å in **NiL1**, **PtL1** and **PdL1**, respectively. On the other hand, the shortest M-O distances of 1.85 Å is calculated for the **NiL1**, which elongates to 1.95 Å in **ZnL1** and 2.00 Å in both **PdL1** and **PtL1**. Therefore, while very small difference of 0.02, 0.03 and 0.04 Å between the M-N and M-O are found for **NiL1**, **PdL1** and **PtL1**, respectively, the significant larger value of 0.11 Å obtained for **ZnL1** implies a higher elongation of the Zn-O bonds with respect to the Zn-N, which might also explain the higher distortion of the corresponding structure.

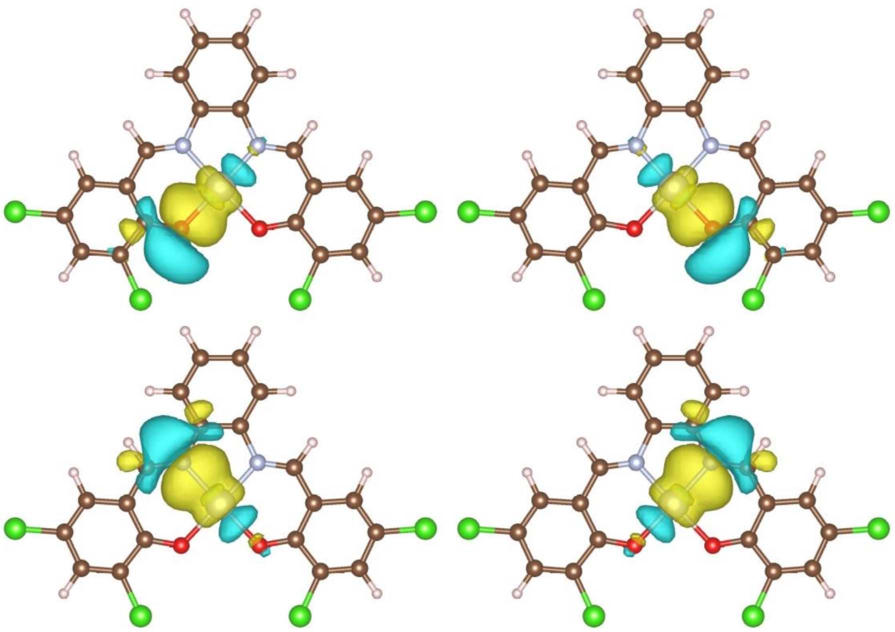

Figure S13. Natural bond order (NBO) representation of the Pt-N and Pt-O bonds in **PtL1**.

4. Calculated ^{1}H-NMR CS values (ppm) of the investigated compounds

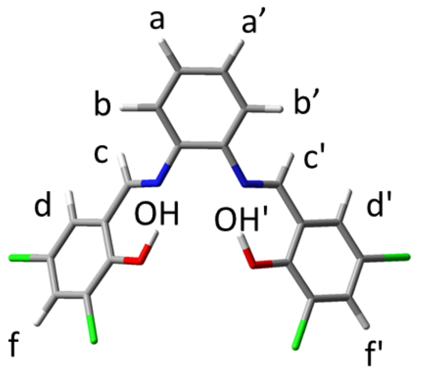

Table S4. H_2**L1**

a	a’	b	b’	c	c’	d	d’	f	f’	OH	OH’
7.981	7.974	7.642	7.764	8.983	9.054	8.023	8.038	8.074	8.047	14.01	14.029

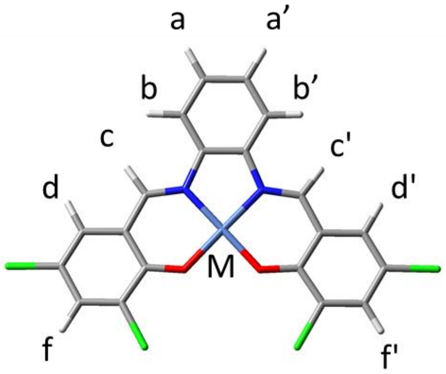

Table S5. **NiL1**

a	a’	b	b’	c	c’	d	d’	f	f’
7.98	7.972	8.536	8.068	9.856	9.848	8.057	8.511	8.084	8.09

Table S6. **ZnL1**

**a**	**a'**	**b**	**b'**	**c**	**c'**	**d**	**d'**	**f**	**f'**
8.054	8.054	8.535	8.535	9.819	9.819	8.088	8.088	8.151	8.152

Table S7. **PdL1**

**a**	**a'**	**b**	**b'**	**c**	**c'**	**d**	**d'**	**f**	**f'**
8.066	8.067	8.647	8.647	9.877	9.878	8.172	8.173	8.212	8.212

Table S8. **PtL1**

**a**	**a'**	**b**	**b'**	**c**	**c'**	**d**	**d'**	**f**	**f'**
8.119	8.12	8.735	8.735	9.934	9.934	8.228	8.229	8.231	8.232

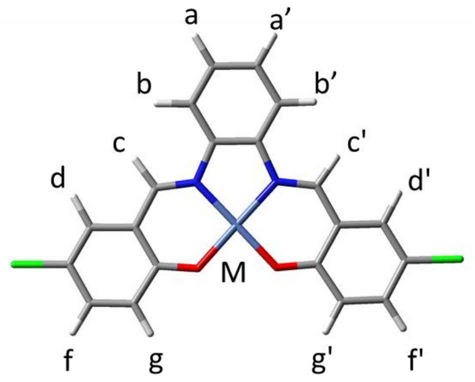

Table S9. **NiL2**

**a**	**a'**	**b**	**b'**	**c**	**c'**	**d**	**d'**	**f**	**f'**	**g**	**g'**
7.936	7.921	8.518	8.487	9.813	9.794	8.064	8.079	7.874	7.878	7.42	7.431

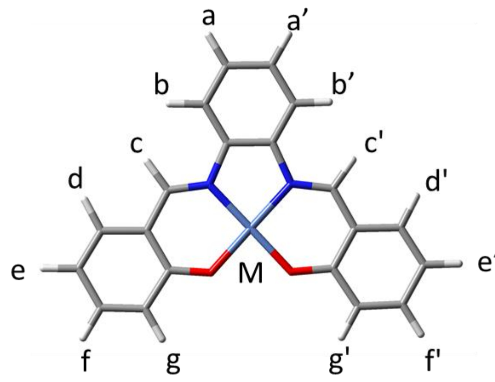

Table S10. **NiL3**

a	a’	b	b’	c	c’	d	d’	e	e’	f	f’	g	g’
7.911	7.893	8.547	8.527	9.916	9.908	8.139	8.155	7.105	7.115	8.004	8.017	7.452	7.457

(NiL1)$_2$

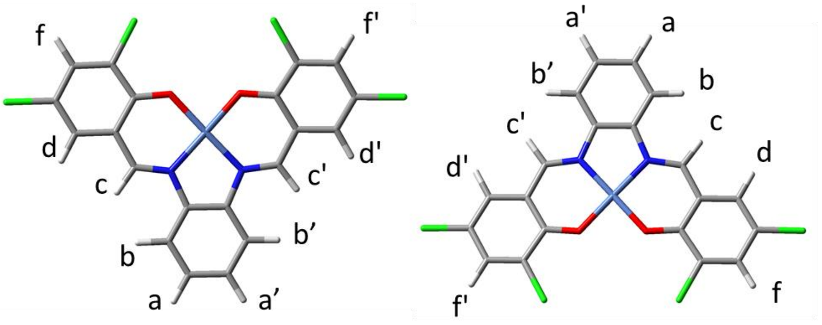

Bottom Top

Table S11. **(NiL1)$_2$**

Bottom

a	a’	b	b’	c	c’	d	d’	f	f’
7.969	8.000	8.177	8.607	9.101	9.553	7.944	6.651	8.211	7.799

Top

a	a’	b	b’	c	c’	d	d’	f	f’
8.000	7.968	8.608	8.163	9.549	9.099	6.65	7.945	7.793	8.207

5. Cartesian coordinates (Å) of the investigate compounds

H_2**L1**

Solvation Free energy = -2870.259775510550 Hartree

C	-3.46919269845463	-1.95405609183031	-0.40718515140320
C	-2.72516945483735	-0.77301826846916	-0.49300257755445
C	-3.13386201684962	0.32685945543738	0.28407926424351
C	-4.24982833948853	0.22504218739446	1.11292382929579
C	-4.96034126584215	-0.95298744525987	1.16965158879403
C	-4.57637866217242	-2.04976271355620	0.41131718240333
O	-1.66472555197690	-0.72140147104125	-1.29484110522392
C	-2.40366155161695	1.58850193891392	0.24166346414892
N	-1.38414758665436	1.74529894193813	-0.50651742847911
N	1.35245372584939	1.77435741481753	-0.52134470156073
C	2.30494580355441	1.58986636043189	0.30385157880836
C	3.08147622115447	0.35528660937075	0.30503683683654
C	2.83070945641663	-0.65688504991102	-0.63991056704127
C	3.62760877681699	-1.80538340066386	-0.60391755789691
C	4.62552127156181	-1.96058158044108	0.33686307207743
C	4.84768348325013	-0.95425594858074	1.26627259731333
C	4.09036247992205	0.19566792969324	1.25339422233476
O	1.87307446283126	-0.55248588464434	-1.55781556722969
Cl	-2.98574110949563	-3.31931998694174	-1.35112040560512
Cl	3.34896835405907	-3.05085921992334	-1.76979846303474
Cl	-6.35485260569340	-1.07663751244184	2.19621942294171
Cl	6.09731484807921	-1.15778626148101	2.45407895591609
C	-0.73153264106199	2.98920107060785	-0.56556708383200
C	0.66906746179606	3.00213476272328	-0.55861609642925
C	1.34602481361274	4.21382539673683	-0.63579716326169
C	0.64324031041732	5.40584282763027	-0.72602224258559
C	-0.74321107379269	5.39335832999810	-0.74190227585260
C	-1.42684461749258	4.18961133408454	-0.66170417048253
H	-4.55065754925216	1.07949369856521	1.70712702147189
H	-5.13538061255199	-2.97519967225363	0.45663465204013
H	-2.76737705175589	2.38790838077384	0.89321156270181
H	2.57742885875620	2.34530669320588	1.04615575641670
H	5.22436530670862	-2.86190094045158	0.34502636396445
H	4.27109541994470	0.98190165193753	1.97645152358367
H	2.42968139412221	4.21293594633426	-0.65222699710728
H	1.18227051416458	6.34247977276271	-0.79915239184729
H	-1.29673740496289	6.32024734278407	-0.82899979085607
H	-2.50985872266867	4.17376169225242	-0.69855573045293
H	1.41916870440296	0.32066596160665	-1.41124347944505
H	-1.27682715080001	0.19128374789022	-1.21208994811103

NiL1

Solvation Free energy = -4377.537628849341 hartree

C	7.31124907652560	4.97919931031026	6.63523759978128
C	6.35522621645466	5.73385556693898	7.36023331165174
C	4.98909556644524	5.43616170767830	7.08733145479734

C	4.61220342852614	4.47511040735718	6.18925070411413
C	5.58815832558053	3.74783184074836	5.49819330776392
C	6.91392099093535	3.99401758086632	5.71166149282610
O	6.64854564010645	6.63998489598675	8.21870513876258
N	9.24656878025588	6.04406921692330	7.61649343406688
C	8.71093640369933	5.19700140946137	6.80481418040511
Cl	3.77341027263417	6.32770771777460	7.93755064716837
Cl	5.08411939272783	2.53172290787113	4.36458177915329
N	10.01390275875748	7.66695751451557	9.42681994682492
C	10.24711554365513	8.41755611231806	10.44928580140176
C	9.25830517673236	9.06791436082739	11.24545004337990
C	7.87907499576308	8.95842193275943	10.93832107705907
C	6.99317056778855	9.68575038666980	11.78373195757109
C	7.43542902590254	10.43474841510302	12.84017093475919
C	8.80577810557340	10.50776946296348	13.11606665021098
C	9.70442196827608	9.84164621332935	12.33320620270305
O	7.40709423206285	8.26421237654644	9.96913235438485
Cl	5.29690606426456	9.60165794335565	11.45046347029496
Cl	9.34214716586197	11.45993397210537	14.46678190235109
C	11.07519529412238	7.08864624725631	8.68109278036401
C	10.65636575236308	6.20006874306396	7.69481492836795
C	11.59192631459809	5.56974739428033	6.88227277206106
C	12.93783683279420	5.82963833370569	7.06421126460912
C	13.35537727336429	6.72154385421454	8.04499125510074
C	12.42894936392679	7.35508622387737	8.85278294102342
H	9.35875352919515	4.58415233890790	6.18456546403675
H	7.67147985850415	3.43584464112471	5.17457969162058
H	3.56169989758813	4.27949130384428	6.01589557437991
H	11.29033357852572	4.88382108837425	6.10269319258205
H	13.66527430249205	5.34014439550015	6.42876387980397
H	12.77464996157348	8.05943365864973	9.59703321782142
H	14.40944886218086	6.93114311020190	8.17730578159551
H	11.27734897249266	8.59040076670695	10.74750250097655
H	10.76647417909433	9.90134510750394	12.53914740893675
H	6.72620014556659	10.96872425387562	13.45970777591836
Ni	8.32913118308864	7.15667028650210	8.80410117937000

ZnL1

Solvation Free energy = -4648.597408894784 Hartree

C	3.60472928400368	-2.07018249834640	-0.00892447905396
C	2.70894101557701	-0.96125530982699	-0.03844470937936
C	3.31287994984005	0.31854772768896	0.13853987127161
C	4.70055007986667	0.43621051368374	0.33556485051355
C	5.50570372741930	-0.66914784685919	0.35103010827745
C	4.95592744114556	-1.93986171776589	0.17774401790077
O	1.45357391498719	-1.17040672682265	-0.2122149964978
C	2.58700744490473	1.56362621495874	0.12149900160609
N	1.32840403127541	1.70052371031074	-0.09218033247204
N	-1.32840966549516	1.70052177440884	-0.09216240131404
C	-2.58701630400398	1.56362336877231	0.12150433487763
C	-3.31288645970178	0.31854326390925	0.13854898751523

C	-2.70894624390343	-0.96126050651026	-0.03843678413420
C	-3.60472667443726	-2.07019388310916	-0.00888621192846
C	-4.95592315009073	-1.93987621773539	0.17779062071721
C	-5.50570394438001	-0.66916195289229	0.3510603206310
C	-4.70055614913981	0.43620063699062	0.33558157273579
O	-1.45358385662043	-1.17040918020105	-0.21223414836291
Cl	2.93463817898630	-3.65459399420876	-0.21853853863539
Cl	-2.93462865766270	-3.65460679738042	-0.21847627166534
Cl	7.22115165307457	-0.50994134987582	0.58471982683004
Cl	-7.22115139357021	-0.50996136772394	0.58475119914021
C	0.70331771880627	2.96596287415976	-0.14336490592476
C	-0.70332392480300	2.96595985990096	-0.14336039539971
C	-1.38762976196290	4.17498763261493	-0.23970153046138
C	-0.69448143263118	5.36881373129238	-0.31212073482261
C	0.69448815052601	5.36881216362960	-0.31212894595925
C	1.38763209600773	4.17498356838066	-0.23971294374127
H	5.12902861455912	1.42269283033188	0.46844935597563
H	5.58838258658207	-2.81833708468522	0.1902345713181
H	3.20136493919244	2.44429150456168	0.31003187267189
H	-3.20137580334597	2.44428916788839	0.31002608786248
H	-5.58837412216694	-2.81835417785530	0.19029946445218
H	-5.12904012878156	1.42268061190658	0.46846745916601
H	-2.46903575944807	4.19339364942900	-0.27599889184819
H	-1.24014591321025	6.30141015219931	-0.38555680458920
H	1.24015449643061	6.30140714312591	-0.38556699408374
H	2.46903804216943	4.19337477852865	-0.27602360282707
Zn	-0.00000701999879	0.12638773312582	-0.20565290036226

PdL1

Solvation Free energy = -2997.064851270417 Hartree

C	-3.52729918210181	-2.11309168109926	0.00015911882061
C	-2.65552968109957	-0.98455860050450	0.00010166979556
C	-3.29137892348474	0.28947125575818	0.00036010328705
C	-4.69844093533273	0.38756362330428	0.00065683348364
C	-5.47821701509518	-0.73229469071856	0.00070604019520
C	-4.89025403063605	-2.00103151943825	0.00045311769278
O	-1.38808666917947	-1.19112832522675	-0.00016546002682
C	-2.58979866406231	1.53921641735935	0.00032147366574
N	-1.31326571862644	1.70778545723951	0.00009593587954
N	1.31325256530745	1.70777846758019	-0.00020792654367
C	2.58978839891690	1.53921994565203	-0.00017152265053
C	3.29138488360957	0.28948654107685	-0.00026152706942
C	2.65553252409213	-0.98453780967473	-0.00047651827857
C	3.52729445321016	-2.11307767405935	-0.00057264420011
C	4.89024955372584	-2.00102130254939	-0.00044623653513
C	5.47821502940108	-0.73228319506818	-0.00022455725910
C	4.69844649536237	0.38758160270350	-0.00014331350526
O	1.38809037073648	-1.19109352895969	-0.00058656148565
Cl	-2.80877952234247	-3.68749707558856	-0.00016732438601
Cl	2.80876371472302	-3.68747571089699	-0.00085084547750
Cl	-7.21036947491850	-0.60471265589277	0.00107469847779

Cl	7.21036812283955	-0.60471231903870	-0.00006047705497
C	-0.69884922757280	2.99306491290857	0.00007104837435
C	0.69884342517690	2.99306434866957	-0.00011193167028
C	1.39119549357860	4.19917857057203	-0.00019959873365
C	0.69446972263958	5.39289740398318	-0.00007299508781
C	-0.69445928962719	5.39290258275054	0.00013881723390
C	-1.39118981280927	4.19918664641177	0.00020718902731
H	-5.15545861819255	1.36966590303316	0.00085035650374
H	-5.50638684008204	-2.89112631528089	0.00048774495319
H	-3.22712530617791	2.41837490614194	0.00050452406617
H	3.22710354952722	2.41838631174325	-0.00005883934792
H	5.50638038526092	-2.89111747639903	-0.00051264513990
H	5.15547302382390	1.36968026733014	0.00002132482463
H	2.47229702477533	4.22438236836181	-0.00038354655598
H	1.24021305035964	6.32793586111222	-0.00014577183182
H	-1.24019561050570	6.32794512207274	0.00023904028146
H	-2.47229159918209	4.22439483443782	0.00036264333923
Pd	-0.00000666603785	0.24768953019296	-0.00022343706178

PtL1

Solvation Free energy = -2988.536546971280

C	-3.51677620080262	-2.14462827613914	0.00026730033954
C	-2.64822816577940	-1.01611831169705	0.00025608693545
C	-3.28106450491613	0.25937953218480	0.00033536202844
C	-4.68894881218150	0.35402136569141	0.00040863762959
C	-5.46790476050464	-0.76608237316197	0.00040450900240
C	-4.88001646457703	-2.03439578241099	0.00033606615490
O	-1.37714147724300	-1.23263106992599	0.00017185478046
C	-2.59031301863279	1.51593601327427	0.00032563147514
N	-1.31255825230728	1.68886815932982	0.00023200336662
N	1.31254681234080	1.68887279000031	-0.00003369264261
C	2.59030265859886	1.51594660629946	-0.00020256831852
C	3.28106000627076	0.25939316034620	-0.00030245253518
C	2.64822888986798	-1.01610755600005	-0.00022537147062
C	3.51678320614578	-2.14461299112929	-0.00033516955570
C	4.88002337596359	-2.03437562873638	-0.00051835591418
C	5.46790387393272	-0.76605833247373	-0.00059727903582
C	4.68894458418790	0.35404430561498	-0.00049068380364
O	1.37714326726997	-1.23262565232972	-0.00006843439058
Cl	-2.79706278525955	-3.71799050661587	0.00017370037562
Cl	2.79707835620577	-3.71797838419882	-0.00023766161795
Cl	-7.19969500375748	-0.63833226993828	0.00047927198518
Cl	7.19969365520639	-0.63830486088730	-0.00083331078738
C	-0.69923729955524	2.97688696631452	0.00018343689013
C	0.69921777832594	2.97689054413259	0.00005130897453
C	1.39177930953606	4.18159267442476	-0.00001448610278
C	0.69434241978782	5.37529116696647	0.00004255562519
C	-0.69437819659427	5.37528837216795	0.00016183866970
C	-1.39180708890704	4.18158541009425	0.00023011384202
H	-5.14711857831815	1.33553611628912	0.00046200085999
H	-5.49521412725443	-2.92498556329331	0.00033273741558

H	-3.22896232644297	2.39298149957581	0.00038810138470
H	3.22894610320444	2.39299635072799	-0.00028924015197
H	5.49522387049337	-2.92496312418146	-0.00059894318230
H	5.14711766834603	1.33555765875618	-0.00055412045926
H	2.47289495585493	4.20624507360978	-0.00011759714321
H	1.23983094295025	6.31045563310405	-0.00001525010043
H	-1.23986988932304	6.31045141499808	0.00019686140819
H	-2.47292334978796	4.20622600669665	0.00031797254701
Pt	-0.00000343234484	0.22480286251991	0.00013226552176

NiL2

Solvation Free energy = -2988.536546971280 Hartree

C	7.31945690925875	4.97578464852267	6.63857989764847
C	6.35545741593102	5.72843119154965	7.35606086983090
C	4.98816092059386	5.45292479507203	7.09447196690651
C	4.61240816152297	4.49296205482205	6.19588259479929
C	5.58401684408987	3.75397741112210	5.50219495238438
C	6.91048859049776	3.99065605846340	5.71448145773873
O	6.64976096836456	6.63801944305665	8.22073736461971
N	9.25076862032540	6.04090155000858	7.61809257965134
C	8.71596008118312	5.18964991194251	6.80686419810923
Cl	5.07464224336089	2.53703429274449	4.36676910372626
N	10.01751896251426	7.66572221846414	9.42576595059934
C	10.25264979969613	8.41869148422014	10.44862200693844
C	9.26421498839989	9.06538332938140	11.24141242739583
C	7.88155577309855	8.95660089269658	10.94691421922850
C	6.98151878947589	9.67085732975583	11.77950305585919
C	7.42608016668895	10.42266769211042	12.83210536674683
C	8.79939908714311	10.50812607829762	13.11139117072416
C	9.70238851867307	9.84705780930949	12.33133415368130
O	7.40592461402006	8.25473157508591	9.97572584713322
Cl	9.33391203097145	11.46781160938520	14.46184491871365
C	11.07838047905355	7.08862550651153	8.68023309324471
C	10.65955126143341	6.19776449371482	7.69514408022154
C	11.59614537746145	5.56796259583588	6.88279073547818
C	12.94218634474201	5.82932920423827	7.06275806341452
C	13.35949955957181	6.72332543287425	8.04161506995455
C	12.43250909283605	7.35679805789020	8.84909862862710
H	9.36594111423017	4.57606310555549	6.18937084573280
H	7.66370739788777	3.42651916049502	5.17681461394192
H	3.56201538310409	4.29833427001485	6.01569924576131
H	11.29463751726606	4.88067164856956	6.10441940629303
H	13.66937964427793	5.33956298540019	6.42727656236168
H	12.77795473968521	8.06305026080485	9.59161397033241
H	14.41333453774018	6.93480307464848	8.17261860432092
H	11.28379273734934	8.59105836879546	10.74435907271483
H	10.76415614288866	9.91454003144680	12.53816398591155
H	6.71925221883135	10.95657660553257	13.45580869471386
Ni	8.33032030095052	7.15318095842730	8.80658644357578
H	5.92323877658680	9.60039258419780	11.56137329616277
H	4.24264442829402	6.02430938903571	7.63307752480118

NiL3

Solvation Free energy = -2539.089686957294 Hartree

C	7.31733277610719	4.98209536204817	6.62454340723020
C	6.34985911161477	5.70756157814420	7.36778377505434
C	4.98454646171364	5.42864827462876	7.10501455453652
C	4.61870712924516	4.49106824633271	6.17496768897751
C	5.58006967274327	3.77048514985882	5.44263751910910
C	6.90474610446603	4.02296989092065	5.67279558276588
O	6.64431017908592	6.59571866302699	8.25767753772332
N	9.24715740962116	6.03276675294849	7.62747577479439
C	8.71037353083219	5.19867805529704	6.79754750899721
N	10.01383344473341	7.65321185077580	9.43829458832056
C	10.24859017097432	8.44310095082008	10.43483008045399
C	9.26548198831158	9.08668765579447	11.23233479847024
C	7.87750751403649	8.92645658099873	10.98253818442558
C	6.98049692448105	9.62647538666814	11.82879815505329
C	7.44019741463620	10.42226346068240	12.84551305745150
C	8.81687592575947	10.57906653491104	13.09005807143750
C	9.70475415695478	9.91652041853317	12.28751296232368
O	7.39775671390187	8.18876575938778	10.03715022395695
C	11.07477401001224	7.07365294221312	8.69532181249180
C	10.65531589909433	6.18760620089413	7.70569796550595
C	11.59230274803181	5.55789734856073	6.89304762116708
C	12.93911997818823	5.81216737713995	7.07745001556774
C	13.35755746475528	6.69672993993157	8.06427773211026
C	12.43064786516827	7.32929761065227	8.87264025469729
H	9.36140066339955	4.60079826174255	6.16562444760999
H	7.66919599970346	3.48501754432346	5.12172411901084
H	3.56506666213668	4.30155041910402	6.00110207355061
H	11.29006847474491	4.87358629030665	6.11230834909817
H	13.66587021814781	5.32169215881218	6.44192679636667
H	12.77832123910365	8.02230613754658	9.62636447723390
H	14.41218780532961	6.89946365344166	8.20291563845059
H	11.28090234520308	8.65092650162648	10.70311115729994
H	10.77288091205358	10.01909167220472	12.44869047608417
H	6.72574301534905	10.94236770779501	13.47431154332413
Ni	8.32456779457070	7.12015727813771	8.83836705730321
H	5.91885499148566	9.50957890286776	11.64678408354621
H	4.23941462659766	5.97962080483752	7.66632989371246
H	9.16179408744440	11.21273805767114	13.89660826229464
H	5.27389186026133	3.03338195841323	4.71201740249234

$(NiL1)_2$

Solvation Free energy = -8755.123182740679 Hartree

C	6.57295356347542	0.10477600092495	2.96895293785144
C	7.90808367626765	-0.18560016275412	2.59232924208160
C	8.92890263547597	0.40304291717137	3.39093441231849
C	8.64700178355120	1.22648384214156	4.44763174338035
H	9.45324819718418	1.66322754890426	5.02288333626101
C	7.31509616806866	1.49787876989036	4.78151261839258

C	6.29378936419232	0.94283034514114	4.06410299272321
H	5.26191772708877	1.15318279692541	4.32002470050979
C	8.92532864021667	2.57489676266904	0.45600503760898
C	7.15171903175061	3.45120294712762	1.74731197946640
H	7.89519127433357	3.97796427231423	2.33859857906809
C	5.79912716062395	3.61512113944057	2.17204490039446
C	4.73416152626325	2.95450428480202	1.51179166601889
C	3.44480224917725	3.11008327610006	2.09293236299568
C	3.23177319455954	3.86218188013753	3.21641423422044
H	2.23519077843170	3.94788283258527	3.63021138214239
C	4.30760342632158	4.51485174316039	3.82799690182585
C	5.56930666592144	4.39627428857036	3.31877500508132
H	6.40663146436942	4.88390568666012	3.80274597869665
C	9.97600242101874	3.17113912431182	1.14333984677303
H	9.79953542896506	3.81759831469649	1.99201786182176
C	11.27610270573538	2.93128376682176	0.74014384964897
H	12.09446022804728	3.39505211810131	1.27621364051592
N	7.54584933759668	2.71924091091543	0.76143284062405
O	4.87113086049230	2.23534891044353	0.45845226213819
C	5.46366765552977	-0.44515946932905	2.25847603177184
O	8.23278864884137	-0.92638328679288	1.59833344258848
C	9.18220861861483	1.74707279621898	-0.63352695954735
C	11.53377305768378	2.09708730821489	-0.34114964668753
N	5.53930198866133	-1.23667770407580	1.24326285059600
H	4.48357302264217	-0.15790272960502	2.62846830780830
N	8.01569105542871	1.22870143984314	-1.25466809002217
C	10.49213805853824	1.50394076683775	-1.03068680540169
H	12.55386206467919	1.90535777483906	-0.64937797642893
C	4.37278026870383	-1.75510680134659	0.62221453040729
C	8.09128964833260	0.43754273840489	-2.27016453945766
H	10.71687236402462	0.86045337591942	-1.87011170591290
C	4.62962554205012	-2.58295451062583	-0.46730725769976
C	3.06285775252493	-1.51240395386506	1.01967278789396
C	6.98197279721333	-0.11237403209394	-2.98062256696976
H	9.07137528359073	0.15060917633374	-2.64046765361741
C	3.57901271042956	-3.17982159403324	-1.15418097592613
N	6.00905469690155	-2.72705584759177	-0.77301006830958
C	2.02124957083570	-2.10594425438909	0.33042752639936
H	2.83811349051630	-0.86908856721093	1.85923588074769
C	7.26109482560499	-0.94984222692374	-4.07622920870124
C	5.64683863435998	0.17746313285983	-2.60360911540187
C	2.27892445349605	-2.94029580409097	-0.75074344173035
H	3.75555698271289	-3.82669195118837	-2.00252928181291
C	6.40316024805328	-3.45813931537747	-1.75955339765481
H	1.00117323831438	-1.91453115598842	0.63889159137292
C	6.23981701112202	-1.50480729127377	-4.79374268583291
H	8.29295967605848	-1.15980885328928	-4.33251110060513
C	4.62599226630355	-0.41130619779054	-3.40217240965300
O	5.32210579257528	0.91788450351690	-1.60939261026229
H	1.46059253907660	-3.40453006741010	-1.28644811201913
H	5.65973295652214	-3.98451497634944	-2.35125969994066

C	7.75572289174378	-3.62150631543773	-2.18455973479133
C	4.90790781891444	-1.23409174981352	-4.45935334923317
C	7.98544519256214	-4.40169898749119	-3.33198450394023
C	8.82068803105990	-2.96107884221319	-1.52416281935724
H	4.10157804228311	-1.67077222861671	-5.03454279217796
H	7.14816214367920	-4.88924166763962	-3.81609715803411
C	9.24698018782817	-4.51942325753691	-3.84180801701223
O	8.68384453897930	-2.24278252128464	-0.47023595296333
C	10.10992335522270	-3.11574330298618	-2.10596280544625
C	10.32279878471749	-3.86684524991361	-3.23011752465610
H	11.31931058634743	-3.95180715083016	-3.64425765076524
Ni	6.44093107413766	1.77724100457144	-0.41222769222190
Ni	7.11404495389978	-1.78536346441488	0.40082321598426
Cl	11.44030419210331	-2.30075136738343	-1.35768359149863
Cl	9.53711098219313	-5.46306917077264	-5.27036822194394
Cl	6.57538459442233	-2.55641619735942	-6.13455616161280
Cl	2.98040732998816	-0.04901510729383	-3.01243985403829
Cl	10.57452247929642	0.04013610122379	3.00189080216104
Cl	6.97982380859936	2.55053670529303	6.12158181145909
Cl	2.11427460647899	2.29505462763703	1.34488627806997
Cl	4.01685597647140	5.45970740271262	5.25560576946814
